# What Would People Think? Social Norms, Willingness to Serve as a Surrogate, and End-of-Life Treatment Decisions

**DOI:** 10.1093/geroni/igaa057.1359

**Published:** 2020-12-16

**Authors:** Rachael Spalding, JoNell Strough, Barry Edelstein

**Affiliations:** West Virginia University, Morgantown, West Virginia, United States

## Abstract

Population aging has increased the prevalence of surrogate decision making in healthcare settings. However, little is known about factors contributing to the decision to become a surrogate and the surrogate medical decision-making process in general. We investigated how intrapersonal and social-contextual factors predicted two components of the surrogate decision-making process: individuals’ willingness to serve as a surrogate and their tendency to select various end-of-life treatments, including mechanical ventilation and palliative care options. An online sample (N=172) of adults made hypothetical surrogate decisions about end-of-life treatments on behalf of an imagined individual of their choice, such as a parent or spouse. Using self-report measures, we investigated key correlates of willingness to serve as surrogate (e.g., decision-making confidence, willingness to collaborate with healthcare providers), and choice of end-of-life treatments. Viewing service as a surrogate as a more typical practice in healthcare was associated with greater willingness to serve. Greater decision-making confidence, greater willingness to collaborate with patients’ physicians, and viewing intensive, life-sustaining end-of-life treatments (e.g., mechanical ventilation) as more widely accepted were associated with choosing more intensive end-of-life treatments. The current study’s consideration of both intrapersonal and social-contextual factors advances knowledge of two key aspects of surrogate decision making—the initial decision to serve as surrogate, and the surrogate’s selection of various end-of-life treatment interventions. Providers can use information about the role of these factors to engage with surrogates in a manner that better facilitates their decision making.

